# Method for reconstructing mortality by educational groups

**DOI:** 10.1186/s12963-021-00264-1

**Published:** 2021-08-26

**Authors:** László Németh, Domantas Jasilionis, Henrik Brønnum-Hansen, Dmitri A. Jdanov

**Affiliations:** 1 Max Planck Institute for Demographic Research, Konrad-Zuse-Straße 1., 18057 Rostock, Germany; 2 Department of Public Health, University of Copenhagen, Øster Farimagsgade 5, postboks 2099, 1014 København K, Denmark

**Keywords:** Educational differences, Old-age mortality, Life expectancy

## Abstract

**Background:**

The lack of classification by educational attainment in death and population exposure data at older ages is an important constraint for studying changes and patterns of mortality disparities by education in Denmark and Sweden. The missing educational distribution of population also restricts analyses aiming at estimating contributions of compositional change to the improvements in national longevity. This study proposes a transparent approach to solve the two methodological issues allowing to obtain robust education-specific mortality estimates and population weights.

**Methods:**

Using nonparametric approach, we redistribute the unknown cases and extrapolate the mortality curves of these sub-populations with the help of population-level data on an aggregate level from the Human Mortality Database.

**Results:**

We present reconstructed and harmonized education-specific abridged and complete life tables for Sweden and Denmark covering 5-year-long periods from 1991–1995 to 2011–2015. The newly estimated life tables are in good agreement with the national life tables and show plausible age- and education-specific patterns. The observed changes in life expectancy by education suggest about the widening longevity gap between the highest and lowest educated for males and females in both countries.

**Conclusions:**

The proposed simple and transparent method can be applied in similar country-specific cases showing large proportions of missing education or other socio-economic characteristics at older ages.

**Supplementary Information:**

The online version contains supplementary material available at 10.1186/s12963-021-00264-1.

## Introduction

Ensuring reliable and timely monitoring of mortality inequalities by socio-economic status has been a challenging task even for highly developed countries. Systematic, comparable across countries and in-time reliable population-level evidence about long-term changes in group-specific mortality is restricted to few Nordic countries having long history of established population registers. In many other countries without possibilities to obtain and link information from population and specific (e.g. educational or income-tax) registers, alternative data approaches have been applied such as census-linkages and census or survey sample mortality follow-ups or using unlinked cross-sectional mortality data. Many countries such as the USA, the UK, Russia, and many other Eastern European countries have been resorting to the so-called cross-sectional unlinked mortality data relying on separate tabulations of census and death records by socio-economic status [1]. This approach has been criticized due to the numerator-denominator bias originating from a possible discrepancy between the sources of information on socio-economic status provided on death and census records [2–4]. Few validation studies revealed substantial biases in the reported information indicated on death records due to higher misreporting probability by proxy informants [4, 5]. It has also been shown that the discrepancy between information sources establishing numerators and denominators may produce notable distortions of group-specific mortality estimates and patterns or even directions of mortality inequalities [4, 6, 7]. On the other hand, the quality of self-reported information at the census may also suffer from various reporting errors.

Nordic countries have been praised for maintaining numerous registers which allow to obtain and merge socio-economic and socio-demographic characteristics about each resident at any time point (at least for the last few decades). Thus, it has been often assumed that the register-based data on mortality by socio-economic group in the Nordic countries are the most precise. However, past and recent studies mention several limitations affecting the quality and scope of group-specific mortality estimates in Denmark and Sweden [8]. One of the most important issues concerns the limited scope of electronic registers with respect to the accounting for numbers and characteristics of foreign-born individuals and identification of socio-economic status of older cohorts. For example, a recent study by [9] reported about over-coverage of migrants in the population register of Sweden. In both Sweden and Denmark, educational registers still do not cover populations at older ages (born before 1915 in Sweden and born before 1922 in Denmark). These problems have important implications for studying mortality inequalities by education and require specific adjustments. Missing information on education at older ages is a particularly important obstacle for studying mortality inequalities in the context of growing influence of mortality and health at older ages. International comparative studies highlight high levels and unfavourable recent changes in mortality inequalities in Sweden, Norway, Finland, and Denmark [10–13]. The persisting or even worsening trends in inequalities have been evolving in the context of strong pro-equitable social policies, suggesting about a specific “Nordic paradox” [14]. Finally, missing education for the deceased and survivors does not allow to identify the magnitude and patterns of compositional shifts and to what extent these transformations influence changes in national longevity.

Many prior studies devoted to monitoring mortality inequalities by education in Sweden and Denmark have been either restricted to a specific age range (e.g. up to the age 89 or 65) or used some arbitrary assumptions. For example, [8] applied the assumption that all education-specific mortality rates above the age 90 are equal to the national (total) mortality rates. Similar approaches have been used in the Danish studies, either applying the same national mortality rates to all educational groups for ages 74+ or applying the education-specific mortality rate ratios observed in 2010 back to the mortality rates in 1987 [11, 12]. To our knowledge, none of the prior studies attempted to reconstruct education-specific composition of the elderly in these countries. Our study aims at filling these evidence and knowledge gaps by proposing a more transparent and statistically grounded general approach to solve the two methodological issues allowing to obtain robust education-specific mortality estimates and population weights for countries having the similar data restrictions.

## Data

The mortality and population data by education for Denmark and Sweden contain a low number of deaths and population with unknown education for younger generations. However, the proportion of such cases is substantially higher at older ages in the earlier periods; therefore, both data corresponding to both countries face similar problems. There is a complete lack of information on education for people born before 1915 in Sweden and born before 1922 in Denmark. As for numerous other studies on mortality differentials in Denmark and Sweden, the provided original (official) data exclude foreign-born populations.

### For Denmark

The available mortality data are a longitudinal register-based dataset for both sexes at least 30 years of age. The data cover more decades starting from year 1991 until the year 2015 aggregated into 5-year-long periods: 1991–1995, 1996–2000, ..., 2011–2015. The baseline always refers to 1 January, and the follow-up stops on 31 December in a given period. Education is grouped into three categories based on the International Standard Classification of Education (ISCED): Low—Primary and lower secondary education (ISCED 1–2); Middle—Upper secondary education (ISCED 3–4); High—Tertiary education (ISCED 5–6). The data are grouped into 5-year-long age intervals: 30–34, 35–39, ..., 85–89, 90+. Note that information on education is not available for people more than 69 years old in 1991, more than 74 in 1996, more than 79 in 2001, more than 84 in 2006 and more than 89 in 2011. Also note that educational level is not systematically registered for immigrants and refugees.

### For Sweden

**Fig. 1:**
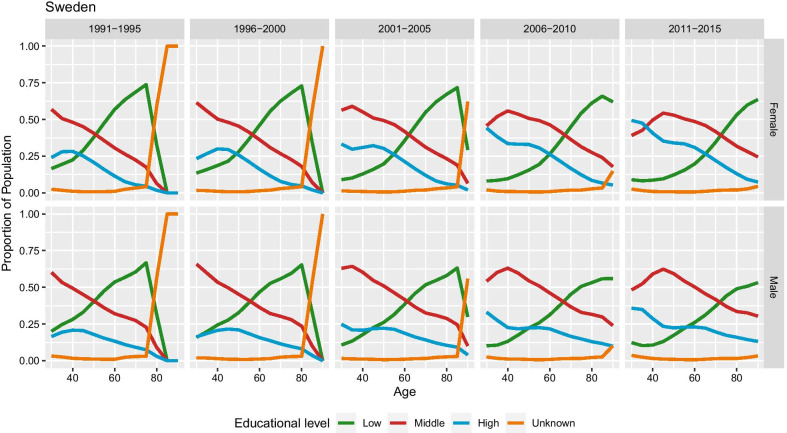
Distribution of population by education, period, and sex in Sweden. The proportion of population with unknown education, designated by orange colour, is higher in the earlier periods and at older ages

The data cover 5-year-long periods between 1991–1995 and 2011–2015. The data are grouped into 5-year-long age intervals: 30–34, 35–39, ..., 85–89, 90+. Education is classified into three categories based on the International Standard Classification of Education: Low—Primary and lower secondary education (ISCED 1–2); Middle—Upper secondary education (ISCED 3–4); High—Tertiary education (ISCED 5–6). Deaths data for ages 85 and 90+ are not available in the first period and for ages 90+ in the second period. The number of deaths at the oldest ages with unknown educational attainment is over 5%, even reaching more than 60% for females at age 90 in the period of 2001–2005. The population data contain slightly lower proportions for unknown cases than that for the death counts, and in addition population is classified by education for more age groups than deaths, e.g. in the first period. Figure 1 presents the Swedish population data showing a high proportion of cases with unknown educational level in the earlier periods of the available data.

## Method

**Fig. 2:**
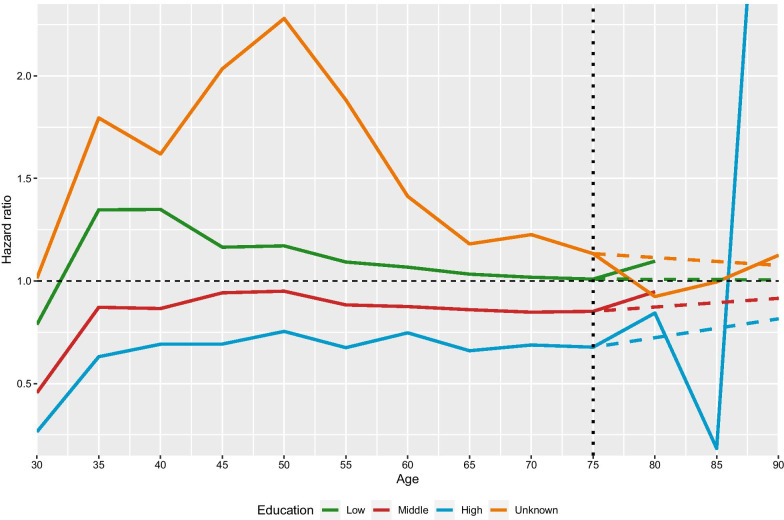
Relationship between mortality of each group compared to the total mortality in the dataset in Sweden in 1991–1995 for females. Each curve is extrapolated between ages 75 and 110, designated by dashed lines, respectively. The extrapolated curves converge to the total mortality and reach the value 1 at age 110

Comparisons of each dataset at the population level to the aggregated country-specific data available in the Human Mortality Database (HMD, [15]) confirmed that the data from different sources are identical. Therefore, we decided to use the information from HMD to classify the unknown cases and extrapolate the mortality curves by educational groups that aggregate to the mortality of the total population. We are going refer to an age group by its lower boundary. Our nonparametric approach consists of the following steps:

(i) First, in a given period we select the last available age at which the number of cases with unknown educational level is below 5%. As an example, in Sweden in the period 1991–1995 at age 75 the proportion of deaths with unknown education is 4% but at age 80 is approximately 56%. Formally, we find the last available age, *a* by solving the following maximization problem:
  $$\begin{aligned}\max _a a \quad \text{ subject }\,\text{ to } \quad a \ge 60 \quad \text{ and }\; \frac{P_u(a)}{P(a)} \le 0.05\end{aligned}$$
  where *P*(*a*) is the population count at age a and *u* denotes the unknown educational attainment. Let $a^*$ denote the optimal solution of the problem above, i.e. the last reliable age in a given period.
  From $a^*$ onwards, the mortality curves are extrapolated to older ages with the assumption that the proportion of each educational level compared to the total mortality is converging to 1, “everyone becomes similar at the end”. At the highest age, $\omega$, the mortality of an educational subgroup is equal to the mortality of the whole population, i.e.
  $$\begin{aligned}\mu _e(\omega )=\mu (\omega ) \qquad \forall e\in \{l,m,h\}\end{aligned}$$
  with *l* corresponding to low, *m* to middle and *h* to high education. For an educational level *e* at age *a* between ages $a^*$ and $\omega$ the hazard can be calculated in the following way:
  $$\begin{aligned}\mu _e(a)=\frac{\frac{\mu _e(\omega )}{\mu (\omega )}-\frac{\mu _e(a^*)}{\mu (a^*)}}{\omega -a}=\frac{1-\frac{\mu _e(a^*)}{\mu (a^*)}}{\omega -a}, \quad a^*\le a < \omega \end{aligned}$$
  For our datasets, we chose $\omega =110$ for multiple reasons. Even though total mortality can be calculated from the education-specific data until age 90 only, life expectancy values are available in the HMD until 110 for each sex irrespective of education. In addition, previous attempts focusing on the subject suggest that convergence of mortality by educational groups does not occur until age 90 [11, 12].
  Figure 2 shows the relationship between the mortality of each educational group compared to the total mortality for Swedish females in 1991-1995. In this period, we extrapolate the hazard ratios from age $a^*=75$. From this age onwards, the extrapolated hazard ratios, indicated by dashed lines, linearly converge and reach the population level hazard with value 1 at age 110.
(ii) Secondly, it is desirable that weighting the mortality for each educational group with its population share should result in the total mortality for the whole population. Please note, according to a sensitivity analysis of educational distributions, educational expansion is rather rigid for the older cohorts in contrast with the more rapid speed of educational expansion in younger cohorts. Based on this observation, to redistribute the population with unknown education at age *a* we assume that the shares between age groups are very rigid. Therefore, we divide the population with unknown educational attainment at age *a*, $P_u(a)$, according to population shares with known educational attainment in the previous age group, in our analysis that is at age $a-5$, and add the result to the already classified cases if there is any. Formally, the population count after redistribution, $P_e^*(a)$ for the education level *e* at age *a* is given by
  $$\begin{aligned}P_e^*(a)=P_e(a)+\frac{P_e(a-5)}{P(a-5)}P_u(a)\delta \end{aligned}$$
  We extrapolate the population shares recursively until age 90 with one condition, denoted by $\delta$, on the proportion of unknown deaths to be redistributed. The magnitude of $\delta$ depends on the death count redistribution in the next step.
(iii) Thirdly, from the extrapolated mortality rates and population counts, the number of deaths after redistribution is given straightforwardly via
  $$\begin{aligned}D_e^*(a)=P_e^*(a)\mu _e\left( a\right) \end{aligned}$$

The sum of deaths in the known educational groups with the unknown together cannot exceed the total number of deaths at a given age. For this reason, we choose the value of $\delta$ in the previous step in such a way that unknown deaths are redistributed in the three educational groups completely.

$$\begin{aligned}D_u^*(a) = D(a) - \sum _{e \in \{l,m,h\}}D_e^*(a) =0\end{aligned}$$

We used DEoptim in R for solving this optimization problem for each sub-population by educational attainment, period and sex.

## Results

**Fig. 3:**
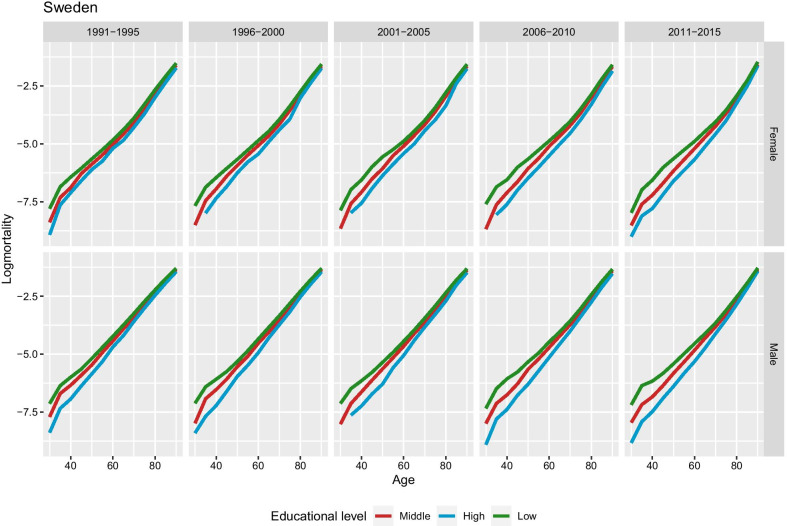
Reconstructed mortality on a logarithmic scale by education, period, and sex in Sweden. Mortality curves fully reconstructed after applying our procedure without irregularities and crossovers at older ages

First and foremost, this method can reconstruct the mortality curve for each subpopulation pertaining to the different educational groups. Previously we had no information in some periods for some ages, especially in the older age groups as mentioned before. Figure 3 presents the estimated mortality curves on a logarithmic scale for different periods, educational level for males and females separately. As intended, the mortality curves do not cross each other; therefore, it reflects that higher education ensures higher life expectancy throughout the whole lifespan. The mortality curve is smooth, and this reflects the regularity of the force of mortality at the oldest old ages. Similar results are obtained for Denmark (figure available upon request).

Based on the estimated mortality values, we build life tables separately for each sub-population with standard assumptions [16]. Life expectancy values for the different educational groups are summarized in Table 1 for ages 30 and 65. We highlight these results to compare remaining life expectancy at age 30 for the whole dataset and at an older age as well, at which mortality is still less-volatile. An additional text file contains the estimated life tables by educational groups in more detail [see Additional file 1: lifetables.txt].

**Table 1:** Estimated remaining life expectancy values in years for each educational group in the first and last periods at ages 30 and 65 for both sexes for Denmark and Sweden

Period	Education	Female		Male	
		30	65	30	65
*Denmark*					
1991–1995	Low	47.7	17.3	42.3	13.7
	Middle	49.9	18.4	44.6	14.5
	High	51.1	19.2	47.4	16.1
	Total	49.4	17.6	44.4	14.3
2011–2015	Low	50.7	19.7	45.9	16.7
	Middle	54.0	21.4	49.5	17.8
	High	56.1	22.8	52.6	19.7
	Total	54.3	21.1	49.6	18.0
*Sweden*					
1991-1995	Low	51.2	19.3	46.1	15.5
	Middle	52.9	20.5	47.8	16.4
	High	54.9	21.9	50.1	17.7
	Total	53.1	19.8	47.8	16.0
2011–2015	Low	52.3	20.4	48.9	17.8
	Middle	54.5	21.5	51.2	18.8
	High	56.7	23.1	53.8	20.4
	Total	55.3	21.6	51.7	18.8

The estimated life tables reflect the general life expectancy increase in both countries regardless of the sub-population. The highest gain was achieved by the highest educated males for both Denmark, 5.2 years and Sweden, 3.7 years of increase at age 30 in the 20 years of the whole analysis period. The record holders are closely followed by highly educated women in both countries with a slightly smaller increase during the same period. Only higher educated males have higher life expectancy than low-educated females for both countries. The gap between highly educated males and females reduced markedly for Sweden (from 4.8 to 2.9 years) but not for Denmark (3.7–3.5). The gap between highly and low-educated groups increased approximately 0.7 years for Sweden and more than 2 years for Denmark with males having a higher disadvantage.

Remaining life expectancy at age 65 increased for Denmark with 3.6 years, 2.7 years for Swedish males and only 1.2 years for Swedish females for the highly educated group. The life expectancy increase at this age for all educational group has lower variability than that at age 30, with Swedish women around 1.1 year, males around 2.5, and somewhat higher for Denmark with 3.3 years of increase except low-educated females (2.4 years only).

**Fig. 4:**
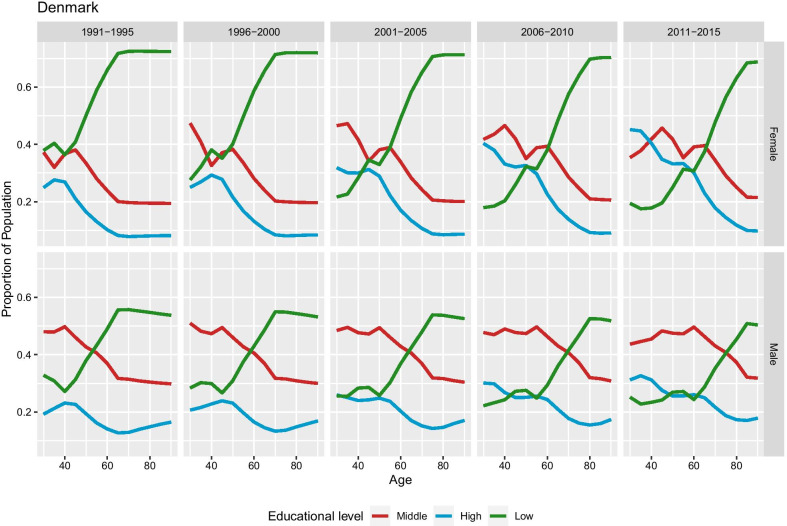
Proportion of population by education, period, and sex in Denmark. According to our assumption, the population shares by educational groups are stable at older ages

Figure 4 presents the reconstructed education-specific population shares for Denmark. The age-specific patterns reflect the underlying assumption regarding the changes in population composition by education at the most advanced ages: (a) there are gradual and not abrupt changes and (b) population composition remain constant after the age 90. Finally, the age- and education-specific proportions are constrained by the fact that education weighted death rates should be equal to the national (total) death rates. The decrease in the low education category is a consequence of the expansion of high and medium-education scenario assumed by our method.

## Discussion

Despite highly developed electronic registers, important gaps in scope and coverage of information on several key characteristics remain in Sweden and Denmark. The lack of classification by educational attainment in death and population exposure data at older ages is an important constraint for comprehensive monitoring of changes and patterns of mortality disparities by education in these two countries. The lacking information has two important implications. First, since overall progress in mortality decline is driven by increasingly advanced ages, the missing information prevents studying the key components and differences of this progress across and within educational groups. Second, missing educational distribution within populations restricts estimating contributions of important compositional changes to improvements in national longevity. This study proposes a transparent nonparametric statistical approach to solve the two methodological issues allowing to obtain robust education-specific mortality estimates and population weights in situations when only aggregated data are available and there is no possibility to run multiple imputation methods and applications [8, 11, 12].

Our approach differs from previously used methods because it does not use strong arbitrary assumptions such as forcing education-specific death rates to be equal to the national death rates after the age 74 or 90 years. We also suggest that standard mortality curve modelling approaches such as applying Gamma-Gompertz or Kannisto models for old-age mortality cannot provide satisfactory solution for obtaining plausible education-specific death rates at higher ages when applied independently for educational groups. This is first of all because one should always consider the relationship between group-specific and total (national) mortality. In order to address this important issue, we used national death rates (covering the total population) from the Human Mortality Database—which itself uses Kannisto-smoothing at the oldest ages—as a reference point assuming that the weighted sum of education-specific mortality death rates is equal to the national death rate within each age group.

Our results highlight recent unfavourable trends in life expectancy disparity by education in Denmark and Sweden. The widening of the life expectancy gap was more pronounced in Denmark and among Swedish males. It is particularly striking that with the exception of Swedish females, a notable widening of disparities also occurred at older ages. Future studies should focus on underlying factors behind these unfavourable changes in the two egalitarian Nordic countries pursuing strong equitable policies. Changing educational composition in both at younger and older cohorts may be an important factor taking into the account rapidly shrinking and increasingly negatively selective lowest education categories. Our study warns that data on socio-economic disparities in mortality should be carefully scrutinized even for the most developed high-income countries maintaining well-established statistical systems. In order to ensure more complete and reliable monitoring of socio-economic mortality disparities in Denmark and Sweden, these countries should dedicate efforts to attempt to fill data gaps on education at old age and establish the reliable education-specific data for foreign-born people.

## Conclusions

Our approach of redistributing cases with unknown educational attainment is a possible method to calculate age-specific life tables and (remaining) life expectancy values for different sub-populations in data with lack of classification. Our simple and transparent method and estimated life tables can stimulate further research on similar country-specific cases showing large proportions of missing education or other socio-economic characteristics at older ages.

## Supplementary Information

**Additional file 1.** Estimated life tables by country, sex and educational attainment.

## Data Availability

The input data were provided by the respective statistical institutes with the condition that only aggregated measures can be published. The derived dataset supporting the conclusions of this article is included within the article and its additional file.

## Abbreviations

ISCED: International Standard Classification of Education
HMD: Human Mortality Database
